# In-laboratory polysomnography as the reference standard in sleep research: a bibliometric overview of human studies

**DOI:** 10.1016/j.clinsp.2026.101157

**Published:** 2026-10-14

**Authors:** Panpan Liu, Mingzhu He, Wenlong Xing, Guiqing Zhang, Shangli Zhang

**Affiliations:** a The First Affiliated Hospital of Shihezi University, Xinjiang, China; b Shihezi University School of Medicine, Xinjiang, China; c Gansu Provincial Hospital, Gansu, China

**Keywords:** Polysomnography, Bibliometric analysis, Sleep

## Abstract

**Background:**

In-laboratory attended Polysomnography (PSG) is the reference standard for objective sleep assessment. Although portable and wearable sleep technologies have advanced, the evolution of PSG research over the past decade has not been systematically characterized.

**Methods:**

Bibliometric data from the Web of Science Core Collection (2015–2025) were retrieved using a search strategy focused on in-laboratory, full-night PSG studies, yielding 462 publications for analysis. VOSviewer, CiteSpace, and Microsoft Excel were used to conduct visual and quantitative analyses of publication trends, country- and institution-level co-authorship, journal distribution, author keywords, and keywords bursts.

**Results:**

Annual publications exhibited a steady increase, with the United States and China as the leading contributors. Prominent institutions included Eindhoven University of Technology and Seoul National University. PSG research was predominantly published in specialized sleep medicine journals. Keyword analysis revealed a stable thematic core encompassing sleep monitoring, sleep staging, and sleep apnea, alongside emerging trends in machine learning, wearable devices, and brain modeling.

**Conclusion:**

Within the corpus of included in-laboratory PSG studies, research outputs demonstrated a sustained trajectory, exhibiting a pattern of parallel development between clinical applications and technical refinement. While PSG remains a foundational methodological pillar in this specific domain, its evolving integration with advanced computational methods underscores a continued commitment to bridging clinical relevance and technological progress within PSG-focused research.

## Introduction

Polysomnography (PSG) is a comprehensive, multimodal technique for the objective assessment of sleep and sleep-related physiological processes. Since its formal standardization, it has served as the reference standard for characterizing human sleep architecture and sleep-related events.^1^ By simultaneously recording neurophysiological, respiratory, and cardiovascular signals under controlled laboratory conditions, in-laboratory PSG provides a uniquely comprehensive characterization of sleep architecture and sleep-related events. This integrative capacity distinguishes PSG from simplified or single-modality approaches. Consequently, PSG has become deeply embedded in sleep medicine, providing the methodological basis for diagnostic criteria, standardized scoring systems, and outcome measures used across both clinical practice and research. It has also served as the cornerstone for defining normative and pathological sleep constructs. Core clinical constructs ‒ including sleep stages, apnea-hypopnea indices, arousal burden, and periodic limb movement metrics ‒ are derived directly from PSG-based measurements. These parameters form the basis of diagnostic criteria, disease severity classification, and outcome assessment across a wide range of sleep disorders, particularly sleep-disordered breathing, hypersomnolence disorders, and parasomnias.

Despite these strengths, PSG is resource-intensive, demanding specialized equipment, trained personnel, and substantial time for data acquisition and manual scoring.^2^ These constraints have motivated ongoing efforts to develop scalable alternatives and complementary approaches.^3^^,^^4^ Concurrent rapid advances in portable monitoring, wearable sensors, and automated analytical algorithms ‒ driven by demands for scalability, accessibility, and cost-effectiveness^5^^,^^6^ ‒ have prompted critical re-evaluations of PSG's centrality. This has fostered a prevailing narrative that laboratory-based PSG may become less necessary or progressively marginalized. However, whether this narrative is substantiated by the contemporary research landscape remains unclear.

Against this backdrop, a critical question emerges: to what extent has in-laboratory PSG actually been utilized in contemporary sleep research? Beyond individual disorders or technologies, it remains unclear whether PSG research has shifted in focus, diversified in application, or consolidated around specific methodological themes over time. A macroscopic, quantitative perspective is therefore essential to map research trends, thematic hotspots, and evolving application domains. Bibliometric analysis provides a systematic approach to address these questions by mapping the structure and evolution of the literature.^7^ While existing bibliometric studies have provided valuable overviews of the broad sleep medicine field, most have not specifically examined the evolution of in-laboratory polysomnography as a distinct technological ecosystem. Accordingly, the present study provides a bibliometric overview of in-laboratory polysomnography as the reference standard in sleep research, aiming to characterize global publication patterns and research themes in human studies over the past decade. To achieve this, the study is guided by the following specific research questions: (i) What are the annual publication trends and growth patterns in-laboratory PSG research? (ii) Which countries and institutions have been the most prolific contributors to this field? (iii) Which journals have served as the primary outlets for publishing PSG research? (iv) What are the major thematic hotspots and evolving research fronts? (v) What emerging topics or bursts of interest have recently appeared in the PSG literature?

## Methods

### *Search strategy and data collection*

The bibliographic data used in this study were obtained from the Web of Science (WoSCC) Core Collection database. Owing to its authoritative status and extensive coverage, the WoSCC is widely adopted as the standard database for conducting bibliometric visualizations and mapping research landscapes.^8^^,^^9^ The search covered publications from 1 January 2015 to 4 December 2025. To ensure terminological rigor, the search string was derived from the Entry Terms listed under the MeSH descriptor “Polysomnography” in PubMed’s MeSH Database. The search formula was TS = (“Polysomnographies” OR “Monitoring, Sleep” OR “Sleep Monitoring” OR “Somnography” OR “Somnographies”). Study selection was conducted in two phases: title/abstract screening and full-text screening. Two investigators (PL and MH) independently screened the records at each stage. In cases where there was uncertainty or disagreement regarding the eligibility of a study, the two investigators discussed the record to reach a consensus. If consensus could not be reached, a third investigator (SZ) was consulted to make the final decision. A total of 1653 records were initially retrieved. After restricting document types to original articles and review articles, 1281 records remained. Subsequently, non-English publications (n = 6), non-human studies (n = 19), and one retracted article were excluded, yielding 1255 records eligible for full-text screening. Then, based on a detailed evaluation of the article title, abstract, or full text, we identified studies employing attended, in-laboratory, full-night polysomnography (Type I PSG) with sleep staging performed according to standardized manual scoring criteria by trained technicians. Studies that utilized automated scoring algorithms were included only if they also met the aforementioned manual-scoring requirement (i.e., manual staging served as the reference standard). Studies were included if PSG was used as a primary data source, reference standard, or validation benchmark. Studies relying exclusively on portable monitoring, home sleep apnea testing, wearable devices, actigraphy, or non-laboratory-based recordings were excluded. After applying these criteria, 462 studies were retained for bibliometric analysis, comprising 420 original articles and 42 review articles. Fig. 1 illustrates the flowchart of the entire selection process.

**Fig. 1:**
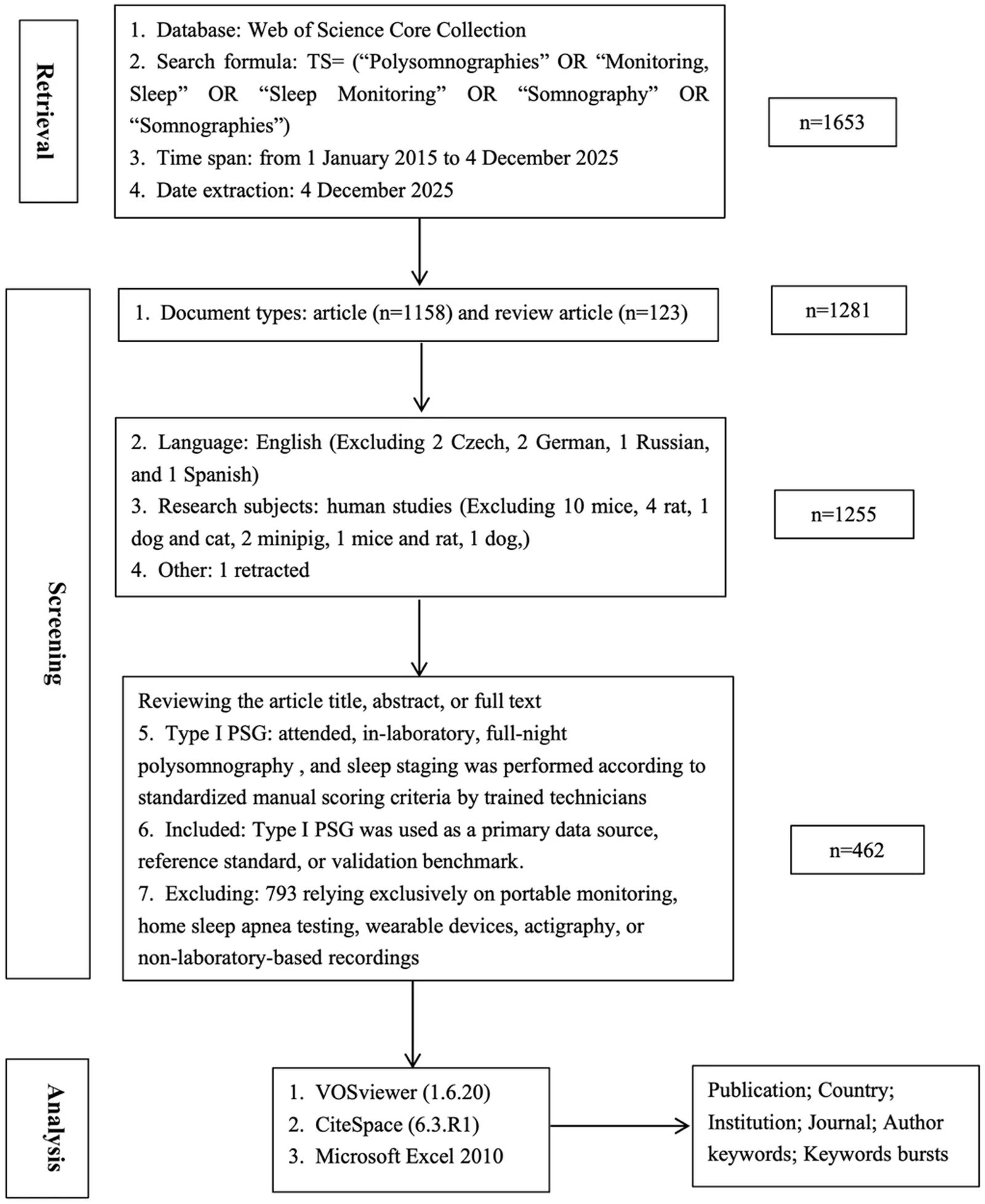
Flowchart of the retrieval, screening, and analysis process.

### *Data analysis*

Bibliometric analysis was conducted using VOSviewer (1.6.20), CiteSpace (6.3.R1), and Microsoft Excel 2010. Full-record data were extracted from the 462 included studies, comprising article titles, authors, abstracts, keywords (author keywords and keywords plus), publication years, source titles (journal name), affiliations (organization, country), document types, languages, and other relevant fields. To examine temporal dynamics, annual publication counts from 2015 to 2025 were compiled and analyzed using Microsoft Excel, providing an overview of historical trends in global research output. VOSviewer was chosen for its advanced visualization capabilities, which enable the creation of detailed and interpretable maps of the research landscape by varying node sizes and colors to represent different elements and relationship.^10^ For the analysis of collaboration networks, co-authorship analysis was applied to extracted fields such as author names, organizational affiliations, and countries, visualizing the structural patterns among key contributing nations and institutions by VOSviewer. A co-authorship network was created to identify leading countries and collaborative networks, with a minimum of 5 documents required for a country to be included. VOSviewer was also used to analyze the distribution of studies across journals, utilizing source title and cited reference data to map the intellectual base of the field. To identify prevailing research themes, author keyword analysis was performed by VOSviewer, revealing high-frequency terms and conceptual clusters. Finally, to detect emerging frontiers and abrupt shifts in focus, CiteSpace was used to identify keywords with significant citation bursts between 2015 and 2025, indicating emerging research trends and topics with rapid increases in attention.^11^

### *Ethics statement*

This study was a bibliometric analysis based exclusively on publicly available literature and did not involve human participants, patient data, or identifiable personal information. Therefore, ethical approval and informed consent were not required.

## Results

### *Publication trends*

A total of 462 studies published between 2015 and 2025 were analyzed. As shown in Fig. 2, the annual number of publications involving in-laboratory PSG exhibited a steady upward trajectory over the study period. Publication output increased steadily from 2015 to 2019, followed by a modest decline in 2020. From 2021 onward, a marked increase was observed. Specifically, the number of publications increased from 18 in 2015 to 64 in 2024, yielding a Compound Annual Growth Rate (CAGR) of 15.1% over the decade. Data for 2025 represent a partial-year count (up to December 4, 2025) and were therefore excluded from the growth rate calculation. Despite minor year-to-year fluctuations, the sustained growth in publication volume reflects continued and increasing scholarly attention to in-laboratory PSG.

**Fig. 2:**
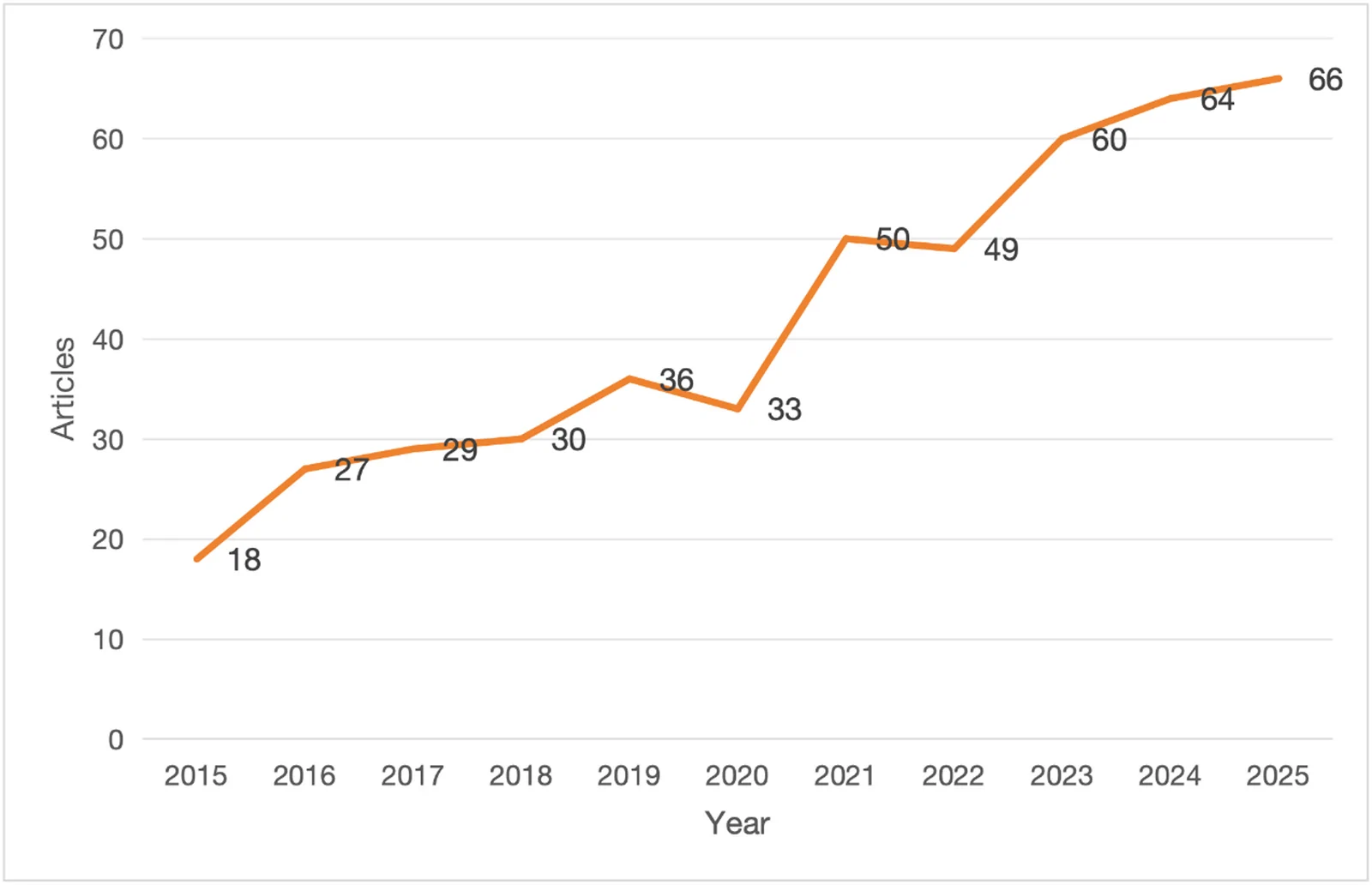
Annual publication trend of PSG from 2015 to 2025. Note: Data for 2025 represents a partial year count (January 1 – December 4, 2025).

### *Countries and institutions*

The country-level co-authorship analysis revealed a globally interconnected research network, with a few core contributors leading the way (Fig. 3). The United States occupied a dominant position in the network, demonstrating extensive collaborative links with multiple countries, including China, Canada, Australia, as well as countries across Europe and East Asia. China formed another major collaborative hub, maintaining strong connections with the United States, the United Kingdom, and Australia. European countries, such as Germany, France, Italy, and the Netherlands, showed dense intra-regional collaboration, while Australia also played an important bridging role within the global network. East Asia and South Asia have assumed growing roles in global PSG research, with countries such as South Korea, India, and Singapore increasing their output and contributing substantially to the field.

**Fig. 3:**
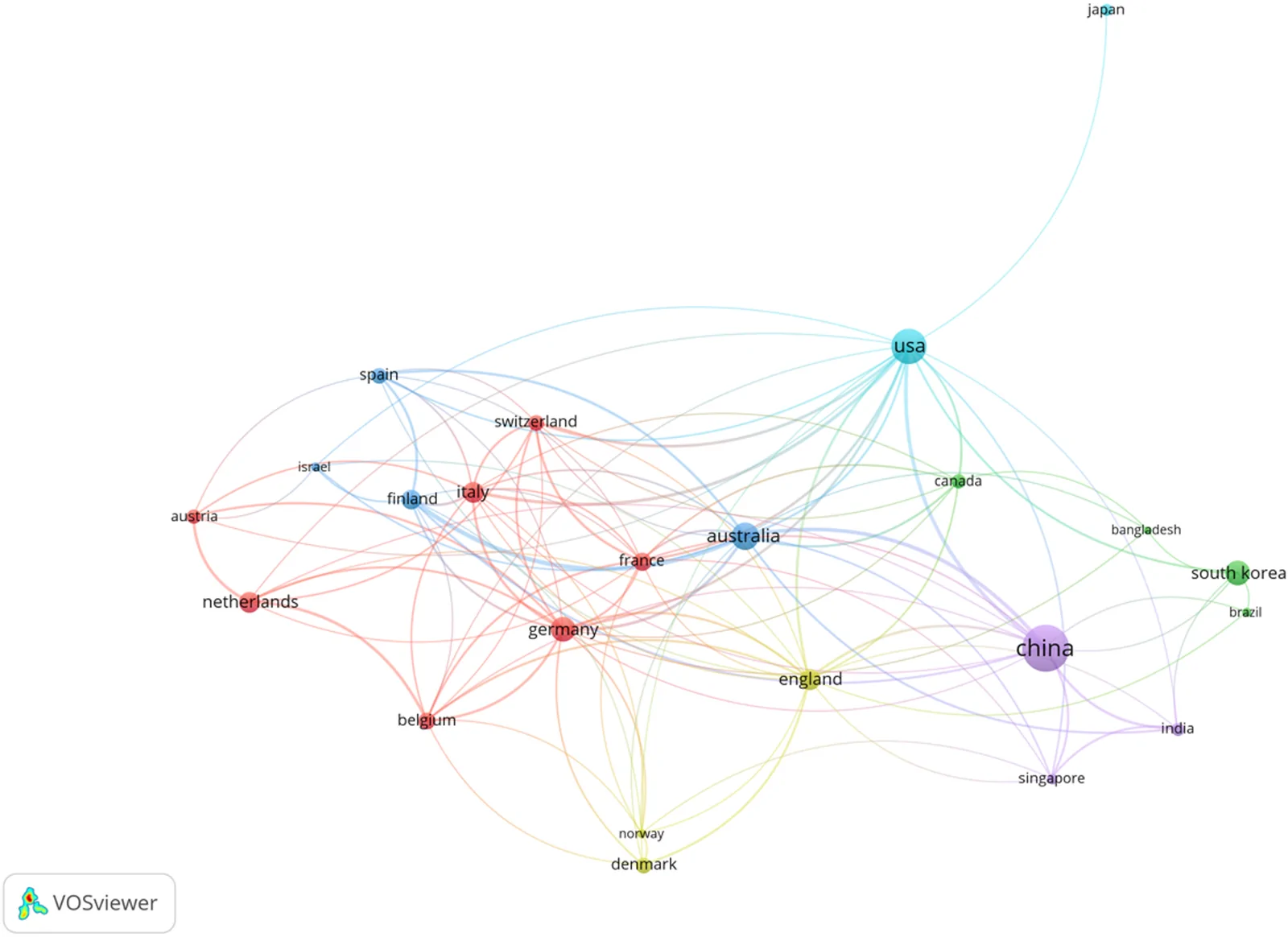
International collaboration network in PSG-based sleep research.The map illustrates country-level co-authorship relationships derived from in-laboratory PSG studies. Node size corresponds to the total link strength (collaboration activity), and link thickness represents the collaboration strength between countries. Colors indicate clusters of countries with similar collaboration profiles.

Of the 462 publications, 312 (67.5%) were single-country studies, while 150 (32.5%) involved international collaboration. This indicates that PSG research is predominantly produced by national teams, although a substantial minority engages in cross-border partnerships, most notably between China and the United States and between Australia and European countries. The most prolific institutions included Eindhoven University of Technology, Seoul National University, the University of Eastern Finland, and The University of Queensland (Table 1). Other top contributors comprised Kuopio University Hospital, Philips Research, and Seoul National University Hospital.

**Table 1:** Top 10 institutions contributing to PSG (2015–2025)^a^.

Organization	Documents	Citations	Total link strength	Country
Capital Medical University	9	122	2	China
Eindhoven University of Technology	16	678	8	Netherlands
Fudan University	9	83	1	China
Harvard Medical School	9	168	1	United States
Kuopio University Hospital	10	191	19	Finland
Philips Research	10	465	8	Netherlands
Seoul National University	16	433	10	South Korea
Seoul National University Hospital	10	234	10	South Korea
University of Eastern Finland	11	191	19	Finland
The University of Queensland	11	157	18	Australia

### *Journal distribution*

Analysis of the journal co-citation network identified a set of core journals constituting the intellectual foundation of PSG-based sleep research during the study period (Table 2). Leading specialized sleep medicine journals, including Sleep, Journal of Clinical Sleep Medicine, Sleep Medicine, and Journal of Sleep Research, ranked highest in citation frequency and total link strength, reflecting their central role in shaping the field. Several review-oriented and subspecialty sleep journals, such as Sleep Medicine Reviews and Sleep and Breathing, also exhibited substantial co-citation metrics. Beyond the core sleep medicine field, high-impact clinical journals in respiratory and cardiovascular medicine (e.g., Chest, American Journal of Respiratory and Critical Care Medicine, European Respiratory Journal, and Circulation) and prominent IEEE journals in biomedical engineering demonstrated exceptionally high link strengths despite contributing minimally to the 462 included publications. This distribution pattern suggests distinct roles within the research ecosystem: clinical journals (e.g., Sleep, Sleep Medicine, Sleep and Breathing, Chest, and Circulation) predominantly publish phenotyping and pathology, where PSG is leveraged to characterize sleep disorders and disease mechanisms,^12^, ^13^, ^14^, ^15^ whereas IEEE journals emphasize methodological innovation and algorithm development, representing the technological frontier of PSG analytics.^16^, ^17^, ^18^

**Table 2:** Top journals in the co-citation network of PSG-related research (2015–2025)^a^.

Journal	Number of citations	Total link strength	Number of Publications
Sleep	1534	23,631	17
Journal of Clinical Sleep Medicine	857	15,054	27
Sleep Medicine	554	10,390	26
Journal of Sleep Research	479	9681	17
Sleep Medicine Reviews	399	7664	4
Sleep and Breathing	306	5673	27
Chest	295	5823	3
American Journal of Respiratory and Critical Care Medicine	283	4996	1
IEEE Transactions on Biomedical Engineering	283	5515	7
IEEE Engineering in Medicine and Biology Magazine	238	5452	0
Sensors	225	5673	17
IEEE Journal of Biomedical and Health Informatics	196	4680	10
Physiological Measurement	195	4213	9
IEEE Transactions on Neural Systems and Rehabilitation Engineering	189	3777	5
PLOS ONE	184	3605	4
Scientific Reports	160	3626	7
European Respiratory Journal	152	3283	1
Biomedical Signal Processing and Control	148	2935	8
Computers in Biology and Medicine	133	2559	5
Circulation	134	2216	0

a ^“^Number of Citations” and “Total link strength” are derived from the co-citation network of references cited by these studies. “Number of Publications” counts only the 462 included PSG studies.

Notably, the same leading sleep medicine journals (Sleep, Journal of Clinical Sleep Medicine, Sleep Medicine) also served as the primary source of the 462 included studies, confirming their dual role as both major producers and the most influential knowledge base in PSG research. This distribution underscores the interdisciplinary nature of PSG-based research, bridging clinical medicine, physiological measurement, and biomedical engineering.

### *Author keywords*

Author keyword analysis was performed to characterize the thematic structure of PSG-related research during the study period (Fig. 4). The thematic structure can be summarized into two overarching domains: a “Stable Core” encompassing the fundamental physiological and clinical applications of PSG, and an “Evolving Methodological Frontier” reflecting the shift toward computational and technological innovation. The density visualization revealed a highly concentrated core centered on “PSG” and “sleep”, underscoring their pivotal position within the research landscape. Closely associated high-density themes included sleep monitoring, sleep stages, and sleep apnea/Obstructive Sleep Apnea (OSA), reflecting the dominant clinical and methodological focus of PSG-based research. Moderate-density clusters emerged around machine learning, automatic sleep staging, and biomedical monitoring, indicating a progressive integration of computational and signal-processing approaches within PSG research. In addition, emerging themes such as wearable devices, home sleep monitoring, and photoplethysmography appeared in peripheral but discernible regions of the density map, indicating expanding interest in alternative monitoring modalities and validation studies that continue to rely on PSG as a reference standard. This dual-structure highlights a stable core of traditional PSG applications alongside a growing body of research exploring advanced analytical methods and novel monitoring technologies within a PSG-centric validation framework.

**Fig. 4:**
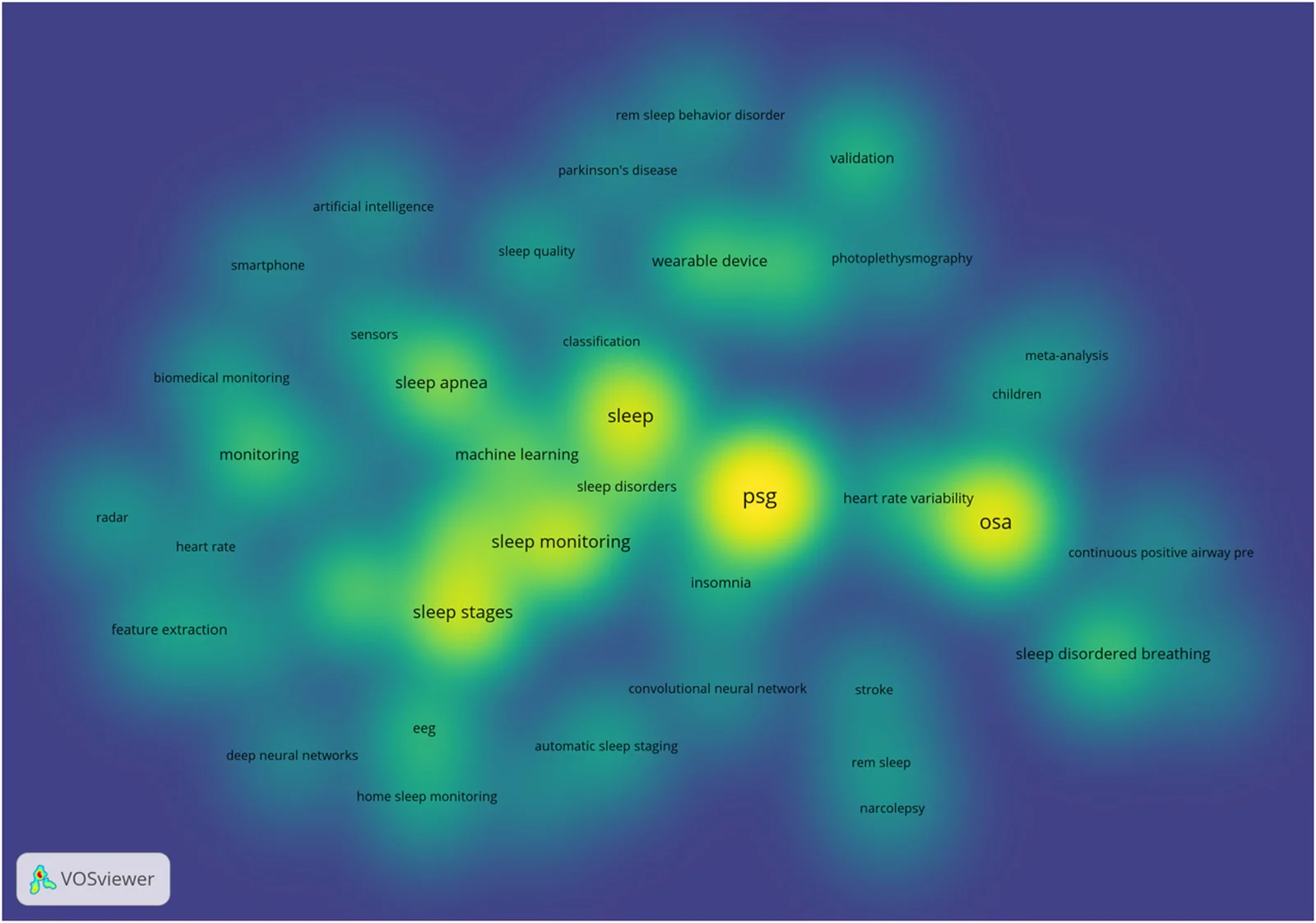
Density visualization of author keywords in PSG research (2015–2025). Warmer colors indicate higher keyword density and stronger co-occurrence.

### *Keywords bursts*

The keyword bursts analysis revealed several significant bursts of research attention in PSG-related studies from 2015 to 2025 (Fig. 5). The most prominent burst occurred in sleep-disordered breathing (2020–2021), with a burst strength of 2.65, reflecting a surge in research attention during this period. Keywords such as central sleep apnea (2015) and sleep apnea (2017) showed early bursts, indicating an initial surge of focus on sleep apnea research. In recent years, keywords like machine learning (2023–2025) and wearable devices (2023–2025) have emerged as high-burst keywords, highlighting the growing interest in integrating AI and wearable technologies into sleep monitoring that employs PSG as the reference standard. Similarly, contactless monitoring and deep neural networks showed bursts in 2022 and 2023, reflecting the increasing application of advanced methods in PSG-based validation studies of novel monitoring approaches. More recent topics such as home sleep apnea testing (2024–2025) and brain modeling (2024–2025) have also shown significant bursts, reflecting an expanding body of PSG-based validation research focused on personalized and remote monitoring solutions.

**Fig. 5:**
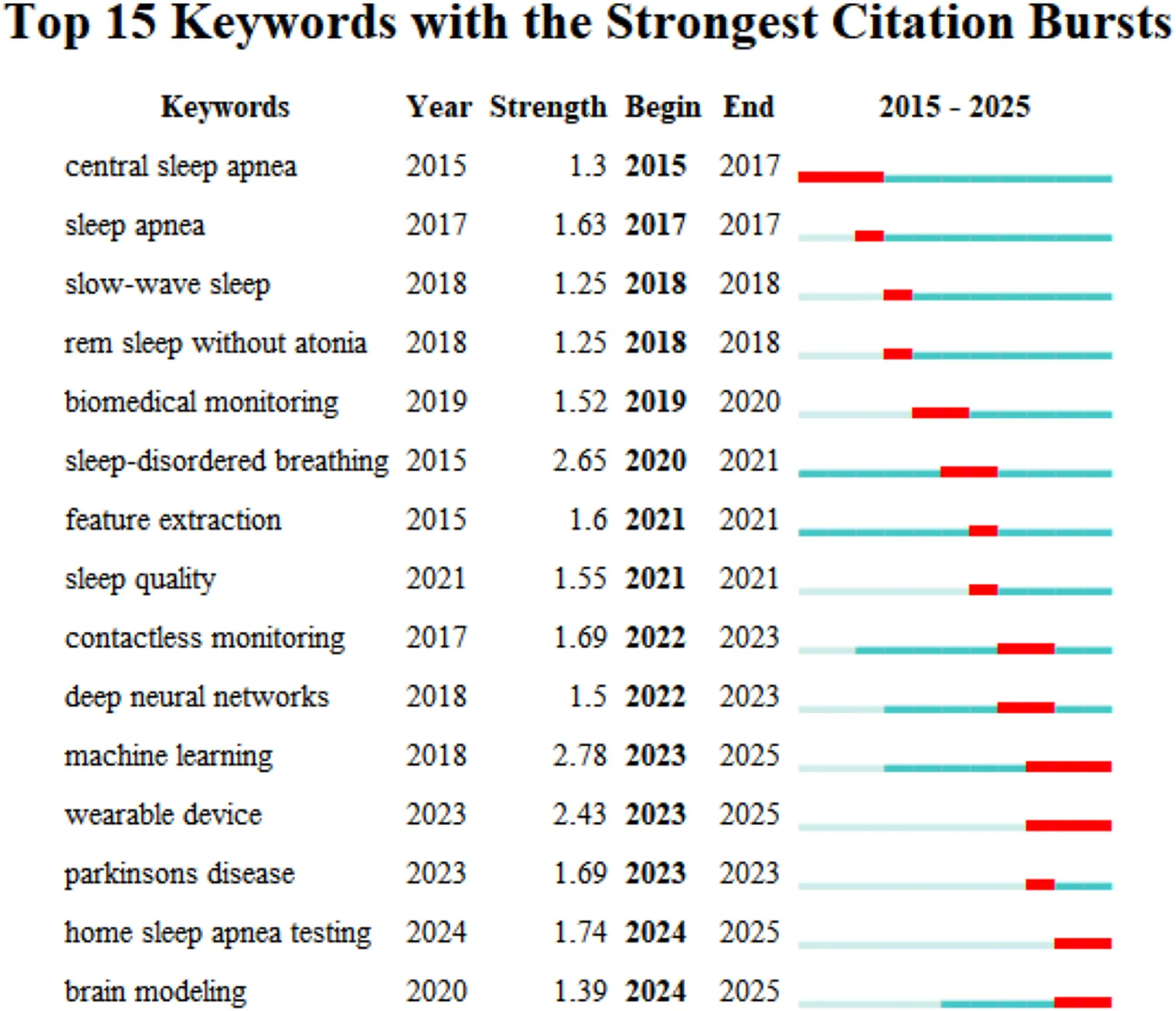
Top 15 keywords with the strongest citation bursts.

## Discussion

As PSG remains the gold standard for sleep assessment, this study provides the first scientometric analysis focusing on in-laboratory PSG as the reference standard in contemporary sleep research. In this study, we collected 462 publications covering the period from 2015 to 2025 for comprehensive visual analysis of PSG. The annual publications showed an overall increase in the number of studies, reflecting the growing interest in PSG and sleep research. Our analysis also revealed a globally interconnected research network, with the United States and China as the dominant hubs, maintaining extensive collaborative links across regions including Europe, East Asia, and Oceania. Key institutions such as Eindhoven University of Technology, Seoul National University, University of Eastern Finland and the University of Queensland have led the way in PSG research. In addition to some important specialized sleep medicine journals, such as Sleep, Journal of Clinical Sleep Medicine and Sleep Medicine, many interdisciplinary journals in respiratory medicine, cardiovascular medicine, and biomedical engineering have also published numerous PSG articles. Furthermore, a stable thematic core centered on PSG and sleep-related disorders has persisted, alongside increasing research interest in machine learning, wearable devices, and contactless monitoring. Notable bursts of research attention were observed in sleep apnea and sleep-disordered breathing, as well as in emerging topics such as home sleep apnea testing and brain modeling within a PSG-centric validation context.

The rising number of PSG publications coincides with​ the increasing global recognition of sleep disorders as a public health priority, as well as ongoing advancements in diagnostic technologies.^19^^,^^20^ However, bibliometric analysis cannot establish a direct causal relationship between these trends. The upward trend in publications also suggests a growing body of evidence supporting the validation and integration of new technologies against the traditional PSG reference standard, underscoring the field’s efforts to adapt to the rapid pace of technological innovation.

PSG represents a globally active area of sleep research, characterized by extensive international collaboration. The United States occupied a central role in the global PSG research network. This prominence aligns with the widespread adoption of clinical practice guidelines and scoring manuals issued by the American Academy of Sleep Medicine (AASM), including the 2023 update to version 3.0 of The AASM Manual for the Scoring of Sleep and Associated Events.^21^, ^22^, ^23^ While these standards may have contributed to the consolidation of PSG research in the United States, this relationship remains correlative rather than causative within the scope of our bibliometric analysis. In addition, highly influential U.S.-based institutions, such as Harvard Medical School, ranked among the top contributors in our institutional analysis, and more than half of the most frequently cited journals in this field are published in the United States, highlighting its role as a hub for international collaboration. China has emerged as another major contributor, forming a distinct collaborative cluster with strong links to the United States, the United Kingdom, and Australia ‒ a transition that underscores China's expanding influence in sleep research, particularly within East Asia. Notably, our keyword burst analysis revealed concurrent surges in topics such as machine learning and brain modeling, which align with the rapid advancement of artificial intelligence technologies in China in recent years.^24^^,^^25^ However, we acknowledge that bibliometric co-occurrence cannot establish a causal link between national AI development and PSG publication growth; this interpretation is offered as the authors’ perspective rather than a direct finding of the analysis. Leading Chinese institutions, including Capital Medical University and Fudan University, ranked among the top contributors in our institutional analysis. Australia also plays an important role in the global PSG research network, acting as a bridge between Eastern and Western research communities. Institutions such as The University of Queensland contribute substantially to this collaborative structure. In Europe, countries including Germany, France, Italy, and the Netherlands demonstrate well-established research networks and strong intra-regional collaboration. Notably, Eindhoven University of Technology and Philips Research, both located in the Netherlands, were among the top contributing institutions, reflecting Europe’s strength in both clinical and technological aspects of PSG research. Although PSG is associated with high costs, substantial technical requirements, and considerable human resources for manual scoring, which may favor concentration of research in economically developed regions such as North America and Europe, our findings indicate that PSG research is not confined to these areas. Instead, a growing number of institutions from East Asia, South Asia, and the Asia-Pacific region, including South Korea, China, India, Singapore, and Australia, are actively contributing to the field. This global participation highlights the expanding geographic diversity of PSG research and suggests a gradual diffusion of advanced sleep research capabilities beyond traditional high-income regions.^26^^,^^27^

PSG research is simultaneously “sleep-core” and “disease-/technology-anchored”. The distribution of publishing journals indicates that PSG research is anchored in a core sleep-medicine publishing ecosystem, while simultaneously spilling over into respiratory medicine, cardiometabolic medicine, and biomedical engineering. This pattern reflects the intrinsic nature of PSG: it is not merely a diagnostic test but a multimodal physiological measurement platform. As a result, PSG-based evidence is routinely interpreted through two complementary lenses ‒ clinical disease phenotyping (e.g., breathing and cardiovascular consequences) and signal-driven methodological development (e.g., automated scoring and feature extraction).^28^^,^^29^ Within this PSG-centric framework, this dual identity explains why high-impact sleep journals remain the central venue for PSG work, yet clinically oriented and engineering journals attract a meaningful share of PSG-related output: the same PSG recording yields both clinically actionable endpoints and methodological advances in algorithmic validation and diagnostic augmentation, dictated by the study’s specific objectives. A second implication of the journal landscape is that the PSG literature is shaped by a blend of clinical consensus and technical standardization. Sleep-focused outlets prioritize methodological rigor in sleep staging and event scoring, while engineering and measurement journals emphasize reproducibility, algorithm benchmarking, and device validation against the PSG reference standard.^30^, ^31^, ^32^ In practice, these two logics reinforce each other: clinical interpretability increases when algorithms are validated against standard PSG outcomes, and technical adoption accelerates when clinical communities recognize standardized scoring frameworks.

The keyword density map points to a stable thematic core around PSG and sleep, with persistent clustering around sleep staging and sleep-disordered breathing (SDB)/OSA. Rather than being “old topics”, these themes remain gravitational centers for three reasons. First, SDB/OSA represents a high-burden condition that is highly PSG-dependent for phenotyping and severity stratification.^33^ Even when simplified testing is used in practice, PSG remains the most comprehensive reference for capturing sleep architecture, arousal burden, hypoxemia patterns, and comorbid movement or parasomnia signals. Consequently, PSG continues to underpin mechanistic research and clinical trial endpoints in this space, which naturally sustains long-term scholarly attention. Second, sleep staging remains central because it is the gateway to nearly all downstream PSG metrics. Many clinical outcomes and physiologic interpretations (respiratory indices, arousal rates, REM-related phenomena, sleep fragmentation) depend on reliable staging. These PSG metrics are not merely physiological markers but serve as vital endophenotypes for cognitive and neuropsychological processes. For instance, sleep architecture (e.g., slow-wave activity and sigma rhythms) is intricately linked to psychomotor development and memory consolidation, while PSG-verified sleep disturbances provide objective biomarkers for understanding the pathophysiology of psychiatric conditions such as psychological trauma and depressive disorders.^13^^,^^34^, ^35^, ^36^, ^37^ Given these high-stakes clinical and psychological implications, the accuracy of sleep staging becomes critical. Consequently, this makes staging both a methodological bottleneck and an innovation target ‒ one reason why automatic scoring and AI methods increasingly appear alongside traditional topics.^38^ Third, the endurance of these cores suggests that the field is not shifting away from classical PSG applications; instead, it is evolving by retooling the same core problems with newer methods. In other words, the “what” of PSG research (sleep staging, SDB) remains stable, while the “how” (analytics, automation, validation pipelines) is rapidly changing.^16^

It is important to emphasize that these technological trends do not represent a general shift away from PSG across the broader sleep research field. Rather, they reflect an expanding body of PSG-based validation research, in which wearable, contactless, and AI-driven tools are evaluated against the established PSG gold standard. AI, wearables, and contactless monitoring are rising, but without “replacing” PSG. The co-occurrence and burst patterns jointly suggest a technology-driven expansion ‒ AI-related terms and wearable/contactless modalities are rising, but typically in a way that still presupposes PSG as the reference standard.^39^ This is best interpreted as a validation-driven innovation cycle, rather than a displacement narrative. A major driver of innovation in sleep monitoring is scalability pressure: laboratory-based PSG is expensive, labor-intensive, and capacity-limited, requiring trained clinicians to spend hours visually inspecting and scoring overnight PSG recordings.^40^ This process is not only time-consuming and tedious but also heavily dependent on clinical expertise, which incentivizes the development of tools that can extend sleep assessment to broader populations while reducing reliance on manual labor. Wearables and contactless monitoring have significantly reduced the pressure of the traditional in-laboratory polysomnography. Looking ahead, a hybrid model may emerge in which wearables serve as high-throughput screening tools, identifying individuals with abnormal sleep patterns who subsequently require confirmatory in-laboratory PSG. This tiered approach could significantly reduce the burden on sleep clinics while ensuring that PSG resources are reserved for complex diagnostic cases. However, scalable tools cannot gain credibility without demonstrating agreement with trusted benchmarks. That benchmark is still PSG, particularly in-laboratory PSG with manual scoring. This explains why wearable devices and contactless approaches often appear at the periphery of the keyword landscape: they are not peripheral in importance, but they are frequently positioned as alternative acquisition modalities whose performance must be mapped back onto PSG-defined constructs. The emergence of “deep neural networks” and “machine learning” bursts likely reflect both increasing computational feasibility and the maturation of datasets suitable for supervised learning. Yet the field’s dependence on PSG-derived labels also highlights a key methodological constraint: model performance is bounded by the quality and consistency of PSG scoring. As automated pipelines proliferate, the community will increasingly need transparent reporting on scoring rules, inter-scorer reliability, and label harmonization across cohorts ‒ otherwise, algorithmic progress may be difficult to compare across studies.^38^ In our view, future research should focus on refining these algorithms to handle edge cases and diverse populations. As automation improves, in-laboratory PSG will become faster and more standardized, allowing clinicians to spend less time on data scoring and more time on data interpretation and patient care.

The recent appearance of topics such as home sleep apnea testing and brain modeling suggests that PSG research is entering a phase of hybridization ‒ linking lab-based gold-standard recordings with broader, more continuous or ecologically valid monitoring strategies. Within the scope of this PSG-focused corpus, this does not necessarily imply that PSG will decline. Instead, based on current trends, we speculate that PSG could serve as a calibration anchor in multi-tiered diagnostic pathways: high-fidelity lab PSG for detailed phenotyping and ground truth generation, complemented by home and wearable modalities for longitudinal tracking.^41^

Bibliometric trends cannot by themselves adjudicate PSG’s long-term clinical role. Recent neuroimaging studies ‒ spanning thalamo-cortical dynamics,^42^ hippocampal connectivity,^43^ and CPAP-related white-matter plasticity^44^^,^^45^ ‒ confirm that in-laboratory PSG remains the reference standard for mechanistic OSA research, providing the physiological substrate underlying our observed thematic clusters. Our work also diverges from the recent OSA neuroimaging bibliometric by Wen et al.^46^ along three dimensions: (i) Scope ‒ we restricted our corpus exclusively to PSG-referenced studies; (ii) Objective ‒ we mapped the evolution of the PSG-centric validation ecosystem rather than neuroimaging broadly; and (iii) Interpretative frame ‒ we conceptualize AI and wearable trends as a “validation-driven innovation cycle” anchored to PSG. Thus, beyond being the first PSG-focused scientometric analysis, this study uniquely characterizes the PSG-as-reference-standard ecosystem. We acknowledge that bibliometric visibility does not equate to clinical superiority; scalable tools still require PSG-derived ground truth. The future of hybrid diagnostic pathways will ultimately be determined by prospective trials, not citation bursts.

Based on current trends, we speculate on two potential future directions: (i) PSG-informed digital phenotyping. AI methods will increasingly move beyond replicating staging toward extracting clinically meaningful phenotypes (e.g., arousal burden, endotype-like respiratory traits, autonomic signatures).^47^ AI can now predict the risk of cardiovascular disease, neurodegenerative disorders, and psychiatric conditions based solely on PSG data. This suggests that the future role of in-laboratory PSG will expand from diagnosing sleep disorders to serving as a comprehensive health risk assessment platform. This aligns with the interdisciplinary journal footprint we observed: respiratory/cardiovascular outlets are natural destinations for phenotype-centric work, while engineering journals focus on algorithmic extraction and validation. (ii) Home and contactless validation at scale. As home monitoring becomes more common, the key research question will shift from “does it match PSG on average?” to “when and for whom does it deviate, and does that deviation matter clinically?” Future PSG-centered studies may therefore emphasize subgroup performance, bias, and generalizability, using PSG as a reference but testing models/devices under real-world constraints. Finally, the prominence of engineering and measurement venues suggests sustained demand for standardized evaluation frameworks ‒ including open reporting of signal quality metrics, artifact handling, and consistent outcome definitions. Interdisciplinary growth is a strength, but it also raises the risk of fragmented terminology. Continued alignment around standardized scoring and reporting conventions will be important to ensure that innovation remains comparable and clinically interpretable.

While this study provides valuable insights into global research trends in PSG, it is important to acknowledge several limitations. First, the analysis was based on bibliometric data from a single database (Web of Science) and limited to English-language publications, which may exclude relevant studies published in other languages or sources. Second, bibliometric data primarily reflect publication and citation patterns, which may not fully capture the clinical impact or quality of research. Third, our search strategy employed a convergent retrieval approach anchored to MeSH Entry Terms rather than exhaustive keyword mapping. While this design prioritized specificity for in-laboratory PSG literature and minimized noise from non-PSG sleep technologies, it may have inadvertently excluded studies using alternative terminologies or non-indexed PSG-related concepts. Future research could benefit from a more diverse data set and include qualitative assessments to better understand the real-world applications and outcomes of emerging technologies.

## Conclusion

This bibliometric analysis provides a comprehensive overview of global research trends in-laboratory polysomnography over the past decade. Despite rapid advancements in portable and wearable sleep monitoring technologies, PSG remains as the reference standard within the specific corpus of laboratory-based sleep research included in this study. Traditional clinical applications, particularly sleep-disordered breathing and sleep apnea, continue to be major focuses, while interdisciplinary approaches involving engineering, signal processing, and computational methods have expanded the thematic scope of PSG research. Emerging interests in artificial intelligence, machine learning, and contactless monitoring signal an evolving trajectory toward technological refinement within this domain. Within the corpus of PSG-based literature, this study demonstrates that research output remains substantial and increasingly interdisciplinary, reflecting a sustained evolution of both clinical applications and methodological innovation. Collectively, these findings outline the developmental trajectory of PSG-based research and may inform future investigations aimed at integrating clinical and technological advancements within this established domain.

## **Data availability**

The Web of Science search query and export date (December 4, 2025) are provided in Supplementary Material S1. Due to Clarivate Analytics licensing restrictions, the full raw bibliographic records cannot be publicly redistributed; however, the derived metadata (e.g., the list of 462 included articles) and analytical parameters are accessible via the supplementary files accompanying this article.

## Authors’ contributions

Panpan Liu: Conceptualized and designed the study and drafted the initial manuscript. Mingzhu He: Contributed to study design and critically revised the manuscript. Wenlong Xing and Shangli Zhang: Performed the data analysis and contributed to interpretation of the results. Guiqing Zhang: Provided critical advice on the study design and interpretation. All authors have read, critically reviewed, and approved the final manuscript.

## Funding

Scientific Research Project of Shihezi University (ZZZC2022079).

## Declaration of competing interest

The authors declare no conflicts of interest.
